# Loneliness and Isolation Versus Wisdom and Compassion During the Pandemic

**DOI:** 10.1093/geroni/igab046.2088

**Published:** 2021-12-17

**Authors:** Dilip Jeste

**Affiliations:** University of California San Diego, La Jolla, California, United States

## Abstract

Our studies of US national-level samples across adult lifespan as well as older adults in California and in Italy’s Cilento region have found a consistently strong inverse correlation between loneliness and wisdom, especially its compassion component. Loneliness and social isolation are associated with worse physical and mental health while the reverse is true for wisdom and compassion. Follow-up of older adults in San Diego during the Covid-19 pandemic showed no change in this pattern. While the effects of the pandemic and the necessary social distancing were heterogeneous, older adults generally handled these stresses better than younger adults, with less loneliness and greater compassion. Our recent studies assessing EEG responses to emotional stimuli as well as alpha and beta diversity in gut microbiome showed opposing biological patterns characterizing loneliness and wisdom. I will also present preliminary data from a compassion training intervention to reduce loneliness among older adults.

